# Immunological Assays used to Support Efficacy of Zika Virus Vaccines

**DOI:** 10.3390/tropicalmed4030097

**Published:** 2019-06-28

**Authors:** Kenneth H. Eckels, Rafael A. De La Barrera, Joseph Robert Putnak

**Affiliations:** Walter Reed Army Institute of Research, 503 Robert Grant Ave., Silver Spring, MD 20910, USA

**Keywords:** Zika, virus, vaccine, assays

## Abstract

In February of 2016, the World Health Organization (WHO) declared Zika virus (ZIKV) a Public Health Emergency of International Concern. This prompted a rapid response from both the private and public sector resulting in the generation of several promising vaccine candidates. In this review, we discuss published scientific efforts associated with these novel vaccines, emphasizing the immunological assays used to evaluate their immunogenicity and efficacy, and support future licensure.

## 1. Introduction

Zika virus (ZIKV) is an arthropod-borne virus (arbovirus) and member of the family *Flaviviridae*, genus flavivirus, which includes other human pathogens such as dengue (DEN), yellow fever (YF), Japanese encephalitis (JE), West Nile (WN) and tick-borne encephalitis (TBE) viruses. All distinguishable ZIKV strains are classified into the African and Asian/American lineages, both of which belong to a single serotype [1]. Similar to other flaviviruses, ZIKV is an enveloped, positive-strand (message-sense) RNA virus with a genome that encodes three structural proteins (C, prM, and E) and seven non-structural proteins (NS1, NS2A, NS2B, NS3, NS4A, NS4B, and NS5). The E, or envelope protein, is the major virion antigen and target of virus-neutralizing antibodies, which are thought to be the best protection correlate. Human infection with ZIKV is often unapparent or mild, similar to a mild case of DEN fever. In the approximately 20% of individuals who develop disease, symptoms include fever, headache, arthralgia, myalgia, conjunctivitis, vomiting, fatigue, and rash [2]. The virus can be detected in serum and other bodily fluids and, perhaps most concerning, it can cross the placenta and infect the fetus causing serious birth defects or death. Additionally, ZIKV infection has been associated with an increased incidence of Guillain-Barré syndrome [3,4]. Prior to the outbreaks in Yap State (2007) and French Polynesia (2013–2014), Zika disease in humans was relatively rare [5]. However, a major ZIKV epidemic in Brazil in 2015 prompted a large-scale private and public response, which resulted in a variety of developers fielding vaccines which are currently being tested in phase 1 and 2 clinical trials. These include DNA, RNA, live-attenuated (recombinant) and inactivated vaccines [6]. 

There are existing licensed vaccines for several other flaviviral diseases including DEN, YF, and JE, and protective levels of neutralizing or binding antibodies have been established for some of these viruses [7,8,9,10], although not for DEN virus (DENV) [11]. DENV vaccine clinical trials currently being conducted may define protective antibody levels for each of the four serotypes. A variety of in vitro assays combined with testing in animal models and human clinical trials are being used to assess ZIKV vaccine immunogenicity and efficacy. We report on those assays published to date (Table 1). Traditional field testing to evaluate efficacy will be difficult for ZIKV due to the sporadic epidemiology of infection and mild disease. Therefore, correlates of protection are discussed that may be used to support vaccine licensure. 

## 2. Assessment of the Humoral Immune Response to ZIKV Vaccines

### 2.1. Functional Assays 

As with other licensed flaviviral vaccines, there is recognition that neutralizing antibodies directed against ZIKV may be used as surrogate markers for vaccine immunogenicity and protective efficacy. This has led many vaccine developers to use virus neutralization as the primary humoral immune response readout. Currently, various assay formats have been employed and a standardized protocol for neutralization testing has not been adopted. Assays include the plaque reduction neutralization test (PRNT), where endpoints are based on visual observation of viral plaques in cell monolayers after inoculation with virus incubated with test serum [20]. Other variations of the neutralization test use ZIKV antisera to measure viral antigen produced by non-neutralized virus in a Vero cell-based enzyme linked immunosorbent assay (ELISA) [16,17,18]. Additionally, detection of foci using immunocytochemistry (focus-reduction neutralization test) [15], and immunofluorescence-based antigen detection using glioblastoma cells [14] have been used in neutralization protocols. A reporter virus particle-based neutralization assay has also been described [12,13,23] for the assessment of immune responses to ZIKV vaccines. This assay uses infectious, subviral particles that contain ZIKV structural proteins and express green fluorescent protein after infection of Raji cells. Virus is detected by flow cytometry. This assay platform was used by Dowd et al. to demonstrate that contemporary South American, Asian, and early African ZIKV strains are similarly sensitive to neutralization by ZIKV human convalescent sera, confirming a single serotype for ZIKV [1]. Additionally, ZIKV neutralization can also be tested using a newly developed, real-time PCR-based neutralization assay for ZIKV, where the neutralization endpoint is measured by a real-time PCR assay [24]. However, without the adoption of a standardized assay format, it will be difficult to directly compare test results among laboratories. Depending upon the assay platform(s) used, variables that could affect virus neutralization titers include (i) the time and temperature of incubation of the virus-serum dilutions, (ii) the time allowed for adsorption of the non-neutralized free virus and immune complexes to the indicator cells, (iii) the origin and passage history of the indicator cell line, (iv) the input virus dose, and (v) whether the viral strain used in the assay is homologous or heterologous to the immunizing strain. One possible solution is the creation of international reference standards, which can be shared among laboratories so that the antibody titers reported are based directly upon the reference standards (see Standardization of Assays, below).

### 2.2. Binding Assays

Although cell-based assays are commonly used to measure virus neutralization, alternative non-cell-based platforms, such as antibody binding tests, are concurrently being used as supplementary and/or confirmatory tests to rapidly and effectively measure the level of anti-ZIKV immunoglobulins (e.g., IgG, IgM) present in serological samples. ELISA assays using whole ZIKV purified virions or recombinant ZIKV E protein have been used for titration of vaccine-generated antibodies [15,16,19,21]. Additionally, to measure quality of antibodies, binding kinetics have been measured in biolayer interferometry (BLI) assays using biotinylated ZIKV E protein and association and dissociation from streptavidin biosensors [25]. A limitation of this assay is the requirement for the use of monomeric E protein. Another antibody quality assay that does not have this limitation is a standard avidity assay that can be used to measure antibody maturation over time. After ZIKV antibodies are adsorbed to live, purified ZIKV, urea is used to dissociate the antibodies. Avidity is measured as a percent of ELISA-bound antibodies measured against non-urea-treated controls [26]. 

## 3. Assessment of the Cell-Mediated Immune Response to ZIKV Vaccines 

Although virus-neutralizing antibody is the primary protection correlate for flaviviruses, measurement of cell-mediated immune responses after vaccination may provide additional useful data. For example, live-attenuated and inactivated vaccines differ in the degree of cytotoxic and helper T-cell responses that they elicit, and this may translate to differences in protective efficacy. 

### 3.1. Intracellular Cytokine Staining 

Peripheral blood mononuclear cells were stimulated with peptide pools that encompassed ZIKV structural proteins. For vaccine responses, proportions of cytokine-positive T cells responding to the peptide pools were summed [13,22].

### 3.2. ELISpot Assays

Peripheral blood mononuclear cells were stimulated with overlapping peptide pools of ZIKV structural proteins. IFNγ secretion was detected by an alkaline phosphatase-conjugated antibody [14,16,17,22].

## 4. Pre-Clinical Assessment of ZIKV Vaccines in Animal Immunization/Challenge Models

Both active and passive immunization have been used to assess protection from ZIKV challenge.

### 4.1. Active Immunization

Both adult mice and non-human primates have been used to assess vaccine immunogenicity and protection against challenge. Adult inbred mice including AG129, Balb/c, SJL, and C57BL/6 have been used for immunization and virus challenge [16,17,20,21,22]. In some studies following challenge with ZIKV, mice were observed for morbidity and mortality. Viremia was also assessed in the vaccinated and control groups of mice to determine the degree of protection.

A new, lethal challenge model using C57BL/6 mice has been developed (D. Smith, Naval Medical Research Center, Frederick, MD, USA, 2019, personal communication). Mice are immunized with a candidate vaccine then treated with anti-interferon monoclonal antibody prior to challenge. This model allows for vaccine responses in immunocompetent mice followed by a lethal challenge. Rhesus macaques and other non-human primates (NHP’s) have also been used for immunization and challenge studies [19,22] and to assess the immunogenicity of Zika and other flavivirus vaccines [27,28].

### 4.2. Passive Immunization

The ability to protect both adult mice and non-human primates using anti-ZIKV immune serum globulin or purified IgG has been demonstrated in passive protection studies. Larocca et al. investigated passive protection in Balb/c mice by purifying IgG from vaccinated mice and intravenously infusing the IgG into recipient mice and challenging with ZIKV soon after. The virus-neutralizing antibody titer of the purified IgG pool could be correlated with protection from viremia after challenge with ZIKV [16]. Abbink et al. showed that purified IgG obtain from rhesus monkeys vaccinated with three different ZIKV vaccines could protect Balb/c mice from viremia after challenge and that protection correlated with serum neutralizing antibody titer [19]. Another study by Muthumani et al. demonstrated that sera from rhesus macaques vaccinated with a DNA vaccine partially protected IFNAR^−/−^ mice after challenge with ZIKV [15]. A similar passive protection study was performed using human vaccinee IgG [18]. Here, Balb/c mice received IgG from subjects that received two doses of ZIKV purified inactivated vaccine and protection correlated with virus neutralization titers. A microneutralization titer of 100 was estimated to be sufficient for conferring partial or complete protection against ZIKV challenge, similar to the titer found in the primate study conducted by Abbink et al. All of these studies demonstrated a positive correlation between virus neutralization titers and protection of mice and NHPs, although the neutralization testing methods varied. 

## 5. Standardization of Assays

As was discussed above for virus neutralization assays, since different laboratories have their own favored assay platforms/SOPs and new assays are continually being developed, the widespread adoption of a standardized assay(s) is unlikely. Nevertheless, in order for serological test results to be compared among laboratories reference standards, especially reference antisera, are needed. To assist in this endeavor, the World Health Organization (WHO) maintains an Expert Committee on Biological Standardization as the scientific body responsible for establishing WHO reference standards. The WHO International Standard (IS) is recognized as the highest reference standard in which antibodies are assigned potencies in International Units (IU). The IS allows for comparison of assays from different laboratories, thereby helping to establish protective antibody levels after vaccination. A WHO collaborative study for testing ZIKV antibodies was organized in 2016 with 19 laboratories from six countries participating in the study. Based on neutralization and enzyme immunoassays, a ZIKV antibody IS was selected and can be requested from the National Institute of Biological Standards and Controls [15] for laboratory use.

The Ebola and Zika outbreaks highlighted the need for International Standards. Although establishing an IS usually requires several years, it would be advantageous to have one in place at the outset of vaccine testing and during pre-clinical development stages [29]. The data could then be bridged to results generated in clinical trials. 

## 6. Complementary Assays That Support ZIKV Vaccine Efficacy 

The development of a safe and efficacious, licensed ZIKV vaccine will require the generation and/or improvement of immunoassays that will be used to evaluate relevant immunological parameters post vaccination. The overall objective of these assays is to help elucidate the nature and types of the immune responses generated by a candidate vaccine, to include the quality and kinetics of the immune response, as well as the longevity of protection associated with the vaccine. Assays designed for this purpose should be validatable, especially if they will be used in later phase 2–3 clinical trials. Validation parameters should include the establishment of an assay cut-off or cut point (positive threshold), linearity, limit of detection (LOD), limit of quantitation (LOQ), specificity, accuracy, and precision. 

Durability of protective immunity is a desirable outcome for any vaccine. It has been found that long-lived plasma cells (LLPCs) are associated with life-long immunity against some viruses following infection and vaccination [30,31]. Mice have been used to study LLPCs that reside in bone marrow. Quantitation of vaccine-generated LLPCs in vaccinated mice could help to establish beneficial long-term immunity with a candidate vaccine.

It will also be important to measure flavivirus cross-reactive antibodies after immunization with a ZIKV vaccine, especially in endemic areas where other flaviviruses are found. This is especially important since it has been postulated that pre-existing DENV immunity may predispose individuals to worsened ZIKV infections. Currently, the best assay for measurement of type-specific and cross-reactive antibody is the virus neutralization assay. Improvements in this assay would be beneficial to sort out homotypic (type-specific) and heterotypic (cross-reactive) antibodies responsible for protection against disease as well as pathogenicity. In one study in Brazil, ZIKV infections and disease did not correlate with DENV immune status [32]. In another study, in vitro antibody-dependent enhancement (ADE) linked to primate pathology has been shown to take place in ZIKV-immune animals that were challenged with DENV-2 [33]. These studies suggest that although prior ZIKV infection (or vaccination) may increase the risk for worsened DENV disease in monkeys, prior immunity to DENV is unlikely to affect the severity of ZIKV infection in humans. However, monkeys are generally not a predictive model for clinical dengue disease but mainly a viremia/infection model, and there are few other studies that show a clear correlation between dengue ADE titers in vitro and the severity of clinical disease. Therefore, large-scale in vitro testing of ZIKV vaccine recipients or the testing of DENV immune populations for infection-enhancing antibodies is unlikely to inform or alter decisions regarding ZIKV vaccine development or deployment. 

Conformational ZIKV epitopes and monoclonal antibodies have been developed that could be used for characterization of vaccine antigens and immune sera. ZIKV-neutralizing monoclonal antibodies generated from plasma cells harvested from volunteers who received a candidate Zika vaccine (ZPIV) were used for epitope mapping (V. Dussupt, Walter Reed Army Institute of Research, Silver Spring, MD, USA, 2019, personal communication). Once fully characterized, these antibodies can be used in antigen binding assays (e.g., ELISA) to demonstrate the presence of relevant epitopes on vaccine antigens, and in antibody competition (competitive binding) assays to determine whether the vaccine is able to elicit antibodies against these potentially important epitopes. In addition to the functional assays (e.g., neutralization tests) aimed at measuring the ability of the ZIKV vaccine to provoke a robust immune response, characterization assays that can assess the overall protective immune response, or efficacy, of the vaccine might be useful. In this regard other antibody effector functions may be important for protection, such as complement-dependent immune cytolysis of infected cells and antibody-dependent cell-mediated cytotoxicity (ADCC). However, unlike virus-neutralizing antibodies, protection correlates (either positive or negative) for antibodies with other effector functions have yet to be established.

## 7. How Assays Can be Used for Licensure

Regulatory views on ZIKV vaccine clinical development strategies have addressed the need for alternatives to traditional phase 3 field trials to gain licensure [34]. Ideally, vaccine effectiveness is evaluated in randomized double-blind trials with a control group receiving a placebo. It is recognized that ZIKV uncertain epidemiology may make this a formidable task and that immune correlates of protection may be helpful to establish efficacy. An immunological correlate of protection (ICP) has been defined as a type and amount of immunological response that correlates with vaccine-induced protection from disease and is considered predictive of clinical efficacy [9]. To date, ICPs are based on humoral immune responses that measure functional (neutralizing) or binding IgG antibodies. For ZIKV vaccine candidates passively transferred antibodies have been found to be protective in animal and human studies [16,18,19] and might serve as an immune correlate. For other flavivirus vaccines including yellow fever, tick-borne encephalitis, and Japanese encephalitis, protective neutralizing or binding antibody levels have been established from primate studies (e.g., using yellow fever) or human efficacy trials of these vaccines [7,8,9,10]. Japanese encephalitis virus purified inactivated vaccine, Ixiaro^®^, is one of these. It was licensed on the basis of demonstrating in vaccinees non-inferior levels of virus-neutralizing antibody compared with antibody titers first established as protective against encephalitis for JE-Vax^®^ (Biken) inactivated vaccine in phase 3 field trials [35].

The U.S. Food and Drug Administration (US FDA) Accelerated Approval provision applies to vaccines that provide meaningful clinical benefit over existing treatments for serious or life-threatening illnesses [36]. Even though ZIKV disease is usually mild, the serious sequelae of Zika congenital syndrome render these provisions applicable for ZIKV vaccines. Registration would be based on data coming from well-controlled phase 1–2 clinical trials that establish a surrogate endpoint, which predicts clinical benefit. For the licensed yellow fever and Japanese encephalitis vaccines, binding or neutralizing antibodies correlate with protection from disease, as was described above. Establishing neutralizing antibody or other immune markers as surrogate endpoints for ZIKV vaccines will be critical for approval under the accelerated approval provisions. Clinical data from studies in endemic and non-endemic areas will also be important to measure the effect of pre-existing antibodies in vaccinated individuals. Testing of sera for baseline evidence of neutralizing antibodies to other flaviviruses that may be found in endemic areas will be critical. Most neutralization assays are highly specific in that they can distinguish among a variety of flavivirus infections and are therefore helpful in evaluating ZIKV vaccine efficacy. Finally, for accelerated approval, post-licensure field studies of vaccine efficacy will be required and can potentially be established by data from case-controlled studies [36]. 

## 8. Use of the US FDA Animal Rule for Establishing Vaccine Efficacy

The US FDA has published guidance for establishing efficacy of preventive vaccines using the “Animal Rule” in “Product Development under the Animal Rule, Guidance for Industry, 2015” [37]. The FDA will rely on animal efficacy data for approval of vaccines using the Animal Rule only when the animal study endpoint is clearly related to the desired benefit in humans, i.e., prevention of morbidity or enhancement of survival. Ideally, the animal model that is chosen should be able to demonstrate progression of disease, signs/symptoms, and immune responses similar to those observed in infected humans when challenged with the etiologic agent. The immune response to the candidate vaccine should be characterized sufficiently so that it can be associated with the desired outcome of disease prevention. The vaccine dose used in animal studies should elicit an immune response that is comparable to that seen in humans. The protective immune response should reflect both antibody and cellular markers similar to those seen in humans.

The rhesus macaque has been used for both pathogenicity as well as vaccine studies and appears to meet most of the Animal Rule criteria for demonstrating vaccine efficacy. In one study, [38], following subcutaneous inoculation, ZIKV RNA was detected in plasma, saliva, urine, and cerebrospinal fluid of all animals. Non-pregnant and pregnant animals remained viremic for 21 days and up to 57 days respectively. The dose of ZIKV used ranged from 10^4^ to 10^6^ plaque-forming units of virus, similar to a dose delivered by a mosquito. Clinical disease included mild weight loss and rash in some of the infected animals. All animals displayed decreased white blood cell counts and elevated liver transaminases. Tests for neutralizing antibodies and T-cell responses were positive. In another study, rhesus macaques infected with a clinical isolate of ZIKV developed fever, viremia, and robust excretion of ZIKV RNA in bodily fluids and the viral antigen was present in organs [39]. Congenital ZIKV syndrome in developing fetuses has been demonstrated in rhesus monkeys infected early in pregnancy [40]. This is an important ZIKV disease manifestation that can be studied in this pre-clinical animal model.

Vaccine studies in rhesus macaques previously cited have established that this non-human primate model may meet the FDA animal rule requirements. Human immune response data from recent phase 1–2 studies appear to follow those results found after immunization of non-human primates [13,18]. Further studies in non-human primates that support efficacy will need to be designed for each type of vaccine being studied. Selection of a challenge strain of ZIKV that causes clinical disease, as well as measurements of vaccine dose responses and demonstration of protection against disease are also required. Such studies must be randomized, blinded and result in data that is statistically significant.

## 9. Human Challenge Studies

Establishing the efficacy of ZIKV vaccines by challenging vaccine recipients with unmodified ZIKV strains has been proposed [41]. The demonstration of protection from illness and infection could be correlated with immune markers such as neutralizing antibodies or cellular immunity. Dengue virus challenge studies have demonstrated that this approach can be done safely in informed human subjects [42]. Measurement of pre-challenge antibodies correlated with protection against dengue-2 virus in these studies. However, with ZIKV long-term virus shedding and the risk for sexual transmission may require that volunteers be quarantined or sequestered until it can be demonstrated that they no longer harbor infectious virus.

## 10. Conclusions

It will be important for ZIKV vaccine developers to develop immunological assays useful for establishing vaccine efficacy. For ZIKV, a flavivirus, it appears that demonstration of virus-neutralizing or binding antibodies are important for establishing vaccine efficacy. Other flavivirus vaccines including YFV, JEV, and TBEV have established surrogate markers of neutralizing and binding antibodies as correlates of disease prevention. Animal studies support these correlates for several ZIKV vaccine candidates by direct challenge of primates as well as transfer of immune sera to mice that are then challenged with ZIKV. Human vaccine studies have also shown that immune sera from vaccinees can similarly protect mice after challenge. The rhesus macaque has been shown to develop ZIKV disease similar to humans and may be useful to establish efficacy based on parameters published by the US FDA. Validated assays will be essential for eventual license of any ZIKV vaccine candidate. Field efficacy testing may be limited due to the sporadic epidemiology of ZIKV and assay correlates will be essential for demonstration of protection against disease.

## Figures and Tables

**Table 1:** Vaccines undergoing development and clinical testing for ZIKV.

Vaccine Developer/Collaborator	Vaccine/Clinical Trial	Pre-Clinical Assays	Clinical Assays	References
Vaccine Research Center (NIAID)	DNA NCT02840487 NCT02996461 NCT03110770	Neutralization (reporter virus particle, focus-reduction, and ELISA MN_50)_); ELISA with subviral particles; immunogenicity in Balb/c and C5BL/6 mice; immunogenicity and efficacy in NHP’s	Neutralization (reporter virus particle); CMI (intracellular cytokine staining)	[12,13]
GeneOne Life Sciences/Inovio Pharmaceuticals, The Wistar Institute	DNA NCT02809443	Neutralization (PRNT_50_); ELISA (E-specific); CMI (intracellular cytokine staining, IFNγ ELISpot); immunogenicity and efficacy in C57BL/6 and IFNAR^-^/^-^ mice; immunogenicity in NHPs	Neutralization (immunofluorescense MN_50_); ELISA; CMI (IFNγ ELISpot); passive protection in IFNAR mice using vaccinee sera	[14,15]
WRAIR, Beth Israel Deaconess Medical Center, NIAID	Inactivated NCT02963909 NCT02952833 NCT02937233 NCT03008122	Neutralization (ELISA MN_50_); CMI (ELISpot, Intracellular cytokine staining); efficacy in Balb/c mice and NHPs; ELISA (E-specific); passive protection in NHPs	Neutralization (ELISA MN_50_); passive protection in mice using vaccinee sera	[16,17,18,19]
Bharat Biotech	Inactivated CTRI/2017/05/008539	Neutralization (PRNT_50_); ELISA (inactivated vaccine-specific); efficacy in AG129 mice; immunogenicity in Balb/c mice; passive immunization in Balb/c mice	NP^1^	[20]
Takeda	Inactivated NCT03343626	NP	NP	
Valneva/EmergentBiosolutions	Inactivated NCT03425149	NP	NP	
Valera/Moderna	mRNA NCT03014089	Neutralization (PRNT_50_, FRNT_50_, reporter virus particle)	NP	[21]
Themis	Measles-vectored NCT02996890	NP	NP	
NIAID/NIH	Live-attenuated (chimeric) NCT03611946	NP	NP	
Janssen	Vectored Ad26 E/M NCT03356561	Neutralization (FRNT_50_); ELISA (E-specific); CMI (IFNγ ELISpot; intracellular cytokine staining); immunogenicity and challenge in Balb/c mice; immunogenicity and challenge in rhesus macaques	NP	[17,19,22]

^1^ NP: Not published.
